# A First Glimpse of the Mexican Fruit Fly *Anastrepha ludens* (Diptera: Tephritidae) Antenna Morphology and Proteome in Response to a Proteinaceous Attractant

**DOI:** 10.3390/ijms21218086

**Published:** 2020-10-29

**Authors:** Eliel Ruiz-May, Alma Altúzar-Molina, José M. Elizalde-Contreras, Jiovanny Arellano-de los Santos, Juan Monribot-Villanueva, Larissa Guillén, Mirna Vázquez-Rosas-Landa, Enrique Ibarra-Laclette, Mónica Ramírez-Vázquez, Rafael Ortega, Martín Aluja

**Affiliations:** 1 Red de Estudios Moleculares Avanzados, Clúster Científico y Tecnológico BioMimic^®^, Instituto de Ecología A.C. (INECOL), Carretera Antigua a Coatepec 351, El Haya, Xalapa 91073, Veracruz, Mexico; jose.elizalde@inecol.mx (J.M.E.-C.); jiovanny.arellano@inecol.mx (J.A.-d.l.S.); juan.monribot@inecol.mx (J.M.-V.); enrique.ibarra@inecol.mx (E.I.-L.); monica.ramirez@inecol.mx (M.R.-V.); 2 Red de Manejo Biorracional de Plagas y Vectores, Clúster Científico y Tecnológico BioMimic^®^, Instituto de Ecología A.C. (INECOL), Carretera Antigua a Coatepec 351, El Haya, Xalapa 91073, Veracruz, Mexico; alma.altuzar@inecol.mx (A.A.-M.); larissa.guillen@inecol.mx (L.G.); mirna.vazquez@inecol.mx (M.V.-R.-L.); rafael.ortega@inecol.mx (R.O.)

**Keywords:** *Anastrepha ludens*, Tephritidae, proteomics, antennae, odorant binding proteins, attractants/traps

## Abstract

*Anastrepha ludens* is a key pest of mangoes and citrus from Texas to Costa Rica but the mechanisms of odorant perception in this species are poorly understood. Detection of volatiles in insects occurs mainly in the antenna, where molecules penetrate sensillum pores and link to soluble proteins in the hemolymph until reaching specific odor receptors that trigger signal transduction and lead to behavioral responses. Scrutinizing the molecular foundation of odorant perception in *A. ludens* is necessary to improve biorational management strategies against this pest. After exposing adults of three maturity stages to a proteinaceous attractant, we studied antennal morphology and comparative proteomic profiles using nano-LC-MS/MS with tandem mass tags combined with synchronous precursor selection (SPS)-MS3. Antennas from newly emerged flies exhibited dense agglomerations of olfactory sensory neurons. We discovered 4618 unique proteins in the antennas of *A. ludens* and identified some associated with odor signaling, including odorant-binding and calcium signaling related proteins, the odorant receptor co-receptor (Orco), and putative odorant-degrading enzymes. Antennas of sexually immature flies exhibited the most upregulation of odor perception proteins compared to mature flies exposed to the attractant. This is the first report where critical molecular players are linked to the odor perception mechanism of *A. ludens*.

## 1. Introduction

The olfactory system in vertebrates and arthropods is finely regulated by soluble proteins (odor binding proteins—OBPs) and odor receptor proteins located in the membrane [1]. OBPs of vertebrates consist of 150–160 amino acids folded in eight β-sheets [2] and OBPs of insects consist of 130–140 amino acids folded in six α-helical domains [3]. The interaction between insects and their environment is strongly mediated by the perception of chemical signals and the fine-tuning modulation capacity to discriminate among thousands of odor sources floating in the air [4]. The study of signal transduction of odor perception in flies (Diptera) is mainly based on knowledge of the OBPs and other soluble chemosensory proteins (CSP) of *Drosophila melanogaster* (Drosophilidae) [1,5]. Proteomics analyses have become a very useful tool for the study of the olfaction system in insects. Pioneering studies have described the presence of OBPs in *D. melanogaster* [6] and several genera of Tephritid flies such as *Ceratitis* [7,8], *Rhagoletis* [9,10], *Bactrocera* [11,12,13] and *Anastrepha* [14,15,16].

*Anastrepha ludens* (Loew) (Diptera: Tephritidae) stands out as one of the most notorious fruit fly pests of citrus and mangoes from Texas to Costa Rica [17,18]. Several behavioral studies have provided clues for the establishment of integrated pest management (IPM) approaches for fruit fly control but more information on olfactory perception to increase the efficacy of attractants and trapping systems is needed [19,20,21]. Unlike the highly effective and selective sexual attractants that have been developed for flies in the genera *Ceratitis* and *Bactrocera*, less effective and generalist food attractants based on hydrolyzed proteins are currently available for IPM strategies against flies within *Anastrepha* [22]. Surprisingly, and considering the importance of *A. ludens* as a pest, limited genomics and molecular studies have been conducted to fully understand critical biological processes related to its control/management, with emphasis on biorational and environmentally friendly schemes. In contrast, several genomics, proteomics and molecular studies related with reproduction, nutrition and odor perception have been reported in two other fruit fly pests of worldwide impact: *Ceratitis capitata* (Wiedemann) and *Bactrocera dorsalis* (Hendel) [7,8,23,24,25,26,27]. Importantly, recent comparative studies suggest partial conservation of the molecular and structural architecture of the olfactory system between tephritids and *Drosophila* [28]. This similarity provides an invaluable resource for embarking on genomics studies on olfactory organs across various economically important genera of tephritid flies. It is well known that females respond more strongly to proteinaceous baits when newly emerged and during the first five to 10 days of adult life, as they need protein to develop their ovaries (and the eggs inside them) and reach sexual maturity [29,30,31]. Additionally, adults of many species, particularly in the case of *Anastrepha*, do not respond equally to the same type of protein-based attractant [32,33], opening a wide window of opportunity to both study antennal structure (including neuronal connections) across species and to dig deeper into the molecular and biochemical mechanisms of odor perception.

Several genes and proteins have been cataloged as potential components of olfactory perception in tephritid flies. However, only a few cases have been associated with the perception of a particular compound. For example, in *C. capitata*, genomics studies lead to the identification of 17 OBPs [7,8]. However, only OBP83a-2 has been associated with perception of (*E,E*)-α-farnesene, a primary compound of male pheromones [34]. Additionally, various genomics studies on different organs/tissues, including antennas, resulted in the identification of several OBPs, chemosensory proteins (CSPs), odorant receptors (ORs), ionotropic receptors (IRs), gustatory receptors (GRs) and sensory neuron membrane proteins (SNMPs) in *B. dorsalis* [12,13,35,36]. Recently, the analysis of head, wings, and antenna in three different physiological adult stages, including newly emerged adults, mature virgin individuals and mated *B. dorsalis* adults, revealed differential gene expression of six OBPs [37]. The functional analysis of OBP99a suggested an association with host location and mating [37]. In the apple pest *Rhagoletis pomonella* (Walsh), pyrosequencing allowed for the identification of seven ORs, two IRs, two GRs, 15 OBPs and also proteins associated with odorant perception, which were possibly related to sympatric speciation [10]. In the case of the walnut infesting fly *Rhagoletis suavis* (Loew), an expressed sequence tag study (EST) was carried out on the antennae and maxillary palps, resulting in the identification of nine OBPs, two CSPs, and one OR [9]. Moreover, in four-day-old adult specimens of *Bactrocera cucurbitae* (Coquillet), 13 OBPs, the odorant receptor co-receptor (Orco), 31 ionotropic glutamate receptors and seven gustatory receptors were identified [11]. Given the strong interest in understanding species differentiation and its association with olfactory perception, a comparative transcriptomics analysis was conducted between *Anastrepha fraterculus* (Wiedemann) and *A. obliqua* (Macquart), two closely related species [15]. An intraspecific and interspecific differential expression analysis of candidate OBPs, showed significant differential gene expression during different reproductive stages in each species [16]. Contrasting differences in gene expression was also observed between these two economically important species.

All the above genomics studies provide the foundation and a powerful in silico tool for proteomics analyses, which can shed further light onto the regulatory mechanisms of olfaction in flies within the family Tephritidae. However, so far, very few proteomics studies have been conducted to understand the biological and ecological foundation of odorant perception in this group of frugivorous insects. In this context, and to understand the perception of methyl eugenol (ME)—a natural phenylpropanoid widely used to attract for monitoring and annihilating *B. dorsalis* males—comparative proteomics using isobaric tags for relative and absolute quantitation (iTRAQ) between ME-responsive and ME-non-responsive males showed the upregulation of 192, and downregulation of 85 proteins [24]. The further molecular characterization of BdorOBP2 by quantitative real-time PCR (qRT-PCR) and RNA interference knock-down analysis suggests its role as a key component in the perception of ME [24]. It thus becomes clear, that the proteomics approach can provide additional, highly useful information when compared to genomics studies. For example, a more accurate identification of authentic molecular players such as abundance, subcellular location, interaction and post-translational modifications of proteins associated with odor perception.

Recently, the commercial attractant CeraTrap^®^, which consists mainly of enzymatic hydrolyzed protein from pig intestinal mucosa, exhibited much superior performance in preferentially trapping females of the pestiferous *A. ludens*, *A. obliqua,* and *A*. *serpentina* (Wiedemann) (Diptera: Tephritidae) when compared to commonly used chemical hydrolyzed protein attractants in fruit orchards [38,39,40]. However, to date, and despite the widespread use of this commercial lure, the active molecule or molecules in CeraTrap^®^ that greatly improve attraction, remain a mystery. In addition, and to the best of our knowledge, the limited information on the molecular and biochemical mechanisms of olfaction, and neuronal circuits severely constrain our understanding of the mode of action of CeraTrap^®^ in the case of flies within *Anastrepha*. To start filling this critical information gap, here we performed a comprehensive proteomics study on the antenna of recently emerged, five-day-old, and sexually mature male and female *A*. *ludens* exposed to water (control) and CeraTrap^®^ volatiles. Based on what we know on the biology of *A*. *ludens*, particularly the need to ingest protein for ovary development, we hypothesized that the strongest expression of odor binding proteins in response to the proteinaceous bait CeraTrap^®^ would be recorded in newly emerged and five-day-old, sexually immature females.

## 2. Results

### 2.1. Morphological Features of Newly Emerged, Five- and 15-Day-Old Antennas

**Figure 1:**
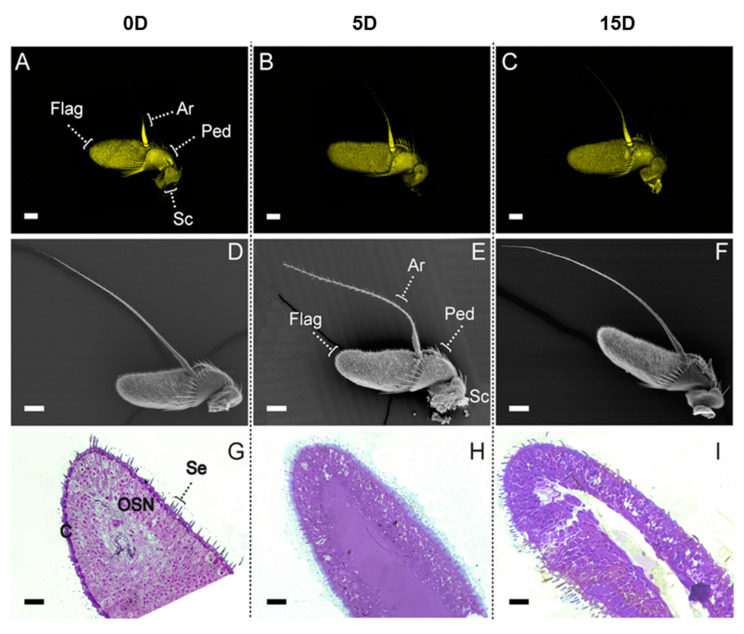
Structural features of *A. ludens* female antennas at three different maturity stages (antennas from newly emerged flies, and from five- (sexually immature) and 15-day-old, sexually mature flies). (**A–C,**) Confocal images. (**D–F**), scanning electron micrograph of the antennas showing shape and segments. (**G–I**), Light microscopy images of one µm thick longitudinal cross-section through the funiculus of the antenna of *A*. *ludens* after staining with toluidine blue. Arista (Ar); flagellum (Flag); pedicel (Ped); scape (Sc); sensilla (Se); cuticle (C); olfactory sensory neuron (OSN). Scale bars (A–F): 100 µm; (G–I): 25 µm.

Our study included three maturity stages of *A. ludens* antennas. In each maturity stage of control flies (exposed to water) or flies exposed—over periods of two or 24 h—to the volatiles emanating from CeraTrap^®^, antennas exhibited great similarities between sexes. Here we concentrated on the morphological characteristics of female antennas exhibiting particularities among developmental stages. Micrographs captured by confocal and scanning electron microscopy depict well-established arista (Ar), scape (Sc), pedicel (Ped) and several sensilla covering the flagellums (Flag) (Figure 1A–F). Importantly, longitudinal cross-sections of antennas exhibited stage-dependent particularities (Figure 1G–I). For example, antennas from newly emerged flies exhibited dense agglomerations of olfactory sensory neurons (OSNs) throughout the internal space (Figure 1G). In contrast, antennas from five (Figure 1H) and 15-day-old flies (Figure 1I) exhibited a clear internal cavity surrounded by OSNs. In addition, antennas from newly emerged flies exhibited notorious cuticle (“C” in Figure 1G) deposition in the external surface compared to five and 15-day-old flies (Figure 1I). Considering these different morphological features, we constructed the proteome profile of each maturity stage in males and female antennas to determine similarities or differences associated with odorant perception.

### 2.2. Proteome Profile of Antennas across Maturity Stages (Newly Emerged, Five-Day, and 15-Day Old Antennas)

**Figure 2:**
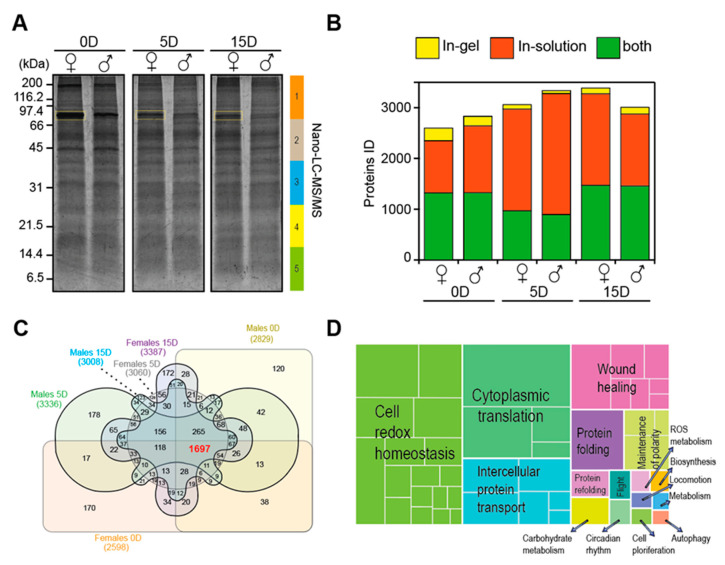
Nano-LC-MS/MS analysis of antennas across different stages of maturity (newly emerged flies, and from five- (sexually immature) and 15-day-old, sexually mature flies) in males and female *A. ludens* flies. (**A**), SDS-PAGE, stained with SYPRO Ruby, of proteins extracted from antennas of newly emerging flies (0D) and antennas of five- (5D) and 15-day-old (15D) flies. Numbers to the left of SDS-PAGE indicate the molecular weight in kDa of the protein markers, while rectangles to the right with various colors indicate the gel sections submitted to tryptic digestion and nano-LC-MS/MS. Yellow boxes indicate the densest protein bands. (**B**), Proteins identified from samples processed either in-gel or in-solution tryptic digestion and subsequent nano-LC-MS/MS analysis. (**C**), Venn diagram of identified proteins showed a core proteome comprising 1697 proteins, indicate in bold red, across males’ and females’ antennas of different maturity stages, newly emerged (0D), five-day (5D), and 15-day (15D). (**D**), Proteins were classified based on gene ontology (GO) enrichment using *Drosophila* protein homologous and David ontology tool (https://david.ncifcrf.gov/). We used the REVIGO web server (http://revigo.irb.hr/) for the visual representation of the clustering of biological processes using abs_log10_pvalue.

Fly antennas compared across maturity stages without exposure to the proteinaceous attractant (i.e., exposed to water), exhibited comparable protein profiles in SDS-PAGE through 200–2.6 kDa. However, a prominent protein band around 70 kDa was detected, distinguished by a yellow rectangle in Figure 2A, and the abundance of which decreased during the maturation process. In-gel and in-solution based tryptic digestions were analyzed by nano-LC-MS/MS and yielded the identification of 4618 unique proteins in the antennas of *A. ludens* across maturity stages. Most of these proteins identified were associated with in-solution based tryptic digestions compared to in-gel digested samples (Figure 2B). However, both approaches contributed to increasing the total number of proteins identified. By comparing the proteome profile of each antenna across maturity stages, we were able to obtain a core proteome consisting of 1697 proteins (Figure 2C).

By means of gene ontology (GO) enrichment and clustering analysis using *Drosophila* homologous proteins, we obtained a background framework of the biological processes associated with the core antennal proteome (Figure 2D, Table S1). A prominent representation of clusters of fundamental biological processes (BP) such as cell redox homeostasis (GO:0045454), intracellular protein transport (GO:0006886) and cytoplasmic translation (GO:0002181), which are essential for sustaining diverse molecular and biological functions in the antennas of flies, became apparent. In addition, clusters including BPs related to wound healing (GO:0042060), protein folding (GO:0006457), carbohydrate metabolism (GO:0005975), flight (GO:0060361) and other functions, also became visible. A closer examination of the wound healing cluster (GO:0042060) exhibited the BP associated with pheromone response (GO:0019236) which grouped several odorant-binding proteins (OBPs) such as OBP99b (Q9VAI6), OBP99a (Q9VAJ4), OBP28a (P54195), OBP Lush (O02372), OBP19a, (Q9VR94), OBP19d (P54192), OBP99c_B (A0A0B4KI20), OBP83a (P54193), OBP69a (P54191), (Q7K084), putative OBP A10 (Q27377), the sensory neuron membrane protein 1 (Snmp1, Q9VDD3), and the universal well-characterized odorant receptor co-receptor (Orco, Q9VNB5, Table 1). Although the clustering analysis did not flush out key BPs associated with the signal transduction of odorant perception, GO enrichment did exhibit BPs such as the cellular homeostasis calcium ion (GO:0006874). Proteins within this BP such as calreticulin (Calr, P29413), calmodulin (Cam, P62152), calcium/calmodulin-dependent protein kinase II (CaMKII, Q00168) and secretory pathway calcium atpase (SPoCk, Q9VNR2) have been linked to the regulation of odorant perception (Table 1). Furthermore, manual scrutiny of our core proteome also provided a catalog of the so-called odorant-degrading proteins (ODPs). The most representative enzymes included glutathione *S* transferases (Gst), cytochrome c oxidases (COX), aldehyde oxidases (AOX), and cytochromes P450 (Cyp, Table 1). We also identified proteins exclusively associated with each maturity stage, and found, for example, that OBP56e (Q7K088), was the only protein identified in mature male flies associated with odorant perception (Table S1).

### 2.3. Comparative Antenna Proteome Profile of A. ludens under Exposure of Ceratrap^®^ by Tandem Mass Tags (TMT) and SPS-MS3 Approach

In this section, we describe the relative abundance of proteins associated with olfaction across samples of flies exposed to water (used as control) and CeraTrap^®^. We note that relative abundance ratios above 1.5 (fold change: CeraTrap^®^/water) indicate upregulation of proteins under CeraTrap^®^ treatments.

#### 2.3.1. Odorant-Binding Proteins (OBPs)

**Figure 3:**
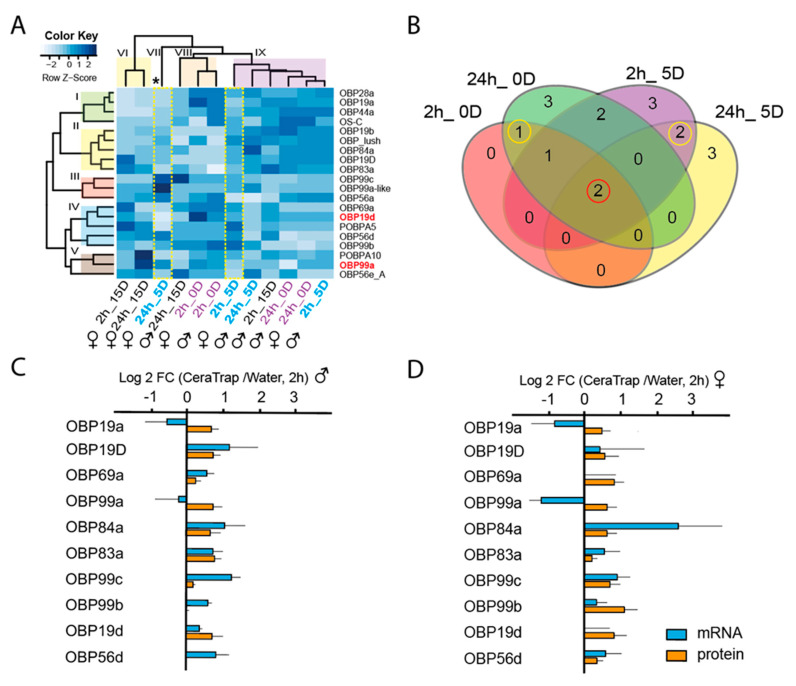
General odorant-binding proteins (OBP) upregulated in antennas of *A. ludens* grouped according to treatments with CeraTrap^®^. (**A**), Heatmap of upregulated OBP in antennas of male and female flies. Our proteomic study included antennas from newly emerged flies (2/24h_0D), and from five- (sexually immature; (2/24h_5D) and 15-day old (2/24h_15D), sexually mature flies exposed to CeraTrap^®^ for two and 24 h. Protein name abbreviations are indicated in the right side of the heatmap. Roman number in left indicates clusters associated with proteins ID and roman numbers on the top specify groups based on protein relative abundance ratios (fold change: CeraTrap^®^/water). Asterisk and dash rectangle indicate the particular upregulation of OBP in 2/24_5D. (**B**), Venn diagram of female immature flies (2/24h_0/5D). Circle in red indicates the common upregulation of two OBP (OBP19d and OBP99a) in all treatments while circles in yellow specified overlap of one OBP in 2/24h_0D and two OBP in 2/24h_5D. Relative mRNA levels (log 2 of Fold change FC mean ± S.E. in blue) and protein (log2 FC mean ± S.E., in orange) in five-day-old male (**C**) and female (**D**) flies antenna treated with CeraTrap^®^ for two hours.

Overall, and importantly, our data clearly show that immature female flies exposed to CeraTrap^®^ were more perceptive at the proteome level and displayed significantly more upregulated OBPs than male flies. The visual representation of our proteomics data by heatmap depicts five hierarchical clusters based on protein annotations (I–V), and four hierarchical groups based on relative abundance ratios (VI–IX, Figure 3A). Five-day-old female flies treated with CeraTrap^®^ over 24 h (24h_5D; group VII) exhibited clear differences in the protein-relative-abundance-profile when compared to the other groups (VI, VIII, and IX) and even when compared to males in the same treatment. In fact, in this sample, it was possible to visualize the most significant upregulation of proteins when comparing all treatments. We could confidently determine the positive upregulation of proteins such as the OBP99c_B, OBP99a-like (A0A0Y0P9P1), OBP56a (Q9V8Y2) from cluster III, OBP56d (Q8SY61) from cluster IV, and OBP99a from cluster V. Additionally, five-day-old female flies exposed over two hours to CeraTrap^®^ (2h_5D) exhibited specific patterns of upregulation compared to other samples in group IX (Figure 3A). Most upregulated proteins included the OBP “Lush” from cluster II and proteins associated with cluster IV such as the putative odorant-binding protein A5 (POBPA5, P54185), OBP56d, and OBP99b. Besides, antennas from newly emerged females exhibited the upregulation of several OBPs under treatment with CeraTrap^®^ for short periods of time (2h_0D). Key examples include the OBP28a and OBP19a from cluster I, OBP Lush from cluster II, OBP69a, OBP19d (P54192), and OBP99b from cluster IV, and OBP99a associated with cluster V. Finally, we could also observe upregulation of some OBPs in mature female flies treated with CeraTrap^®^ (2/24 h _15D) (Table S2).

We further compared upregulated OBPs across samples and observed an overlap of two OBPs, OBP19d and OBP99a (Figure 3B). Furthermore, OBP19a was exclusively upregulated in newly emerged immature flies while OBP99c_B and POBPA10 in antennas of five-day-old flies (Figure 3B), in both cases exclusively in females (Table S2). The upregulation of specific OBPs at particular fly developmental stages, suggests individual CeraTrap^®^ perception mechanisms at each maturity phase.

To underpin our proteomics data, we determined the gene expression of some OBPs determined in our proteomics analyses. We analyzed five-day-old male and female flies exposed to CeraTrap^®^ over two hours since the most significant upregulation of proteins was associated with these flies. Most OBPs mRNA upregulation was directly correlated with protein abundances, except for OBP19a and OBPP99a which exhibited negative regulation in male and female antennas (Figure 3C,D). However, gene expression among the OBPs was significantly different (ANOVA, F_9,59_ = 3.146, *P* = 0.006), but in this case sex played no significant role (F_1,59_ = 0.710, *P* = 0.404) (Table S3). In the case of the relative abundance of OBPs, no differences were found among the protein groups (F_9,59_ = 0.916, *P* = 0.521) and between sex (F_1,59_ = 2.61, *P* = 0.114). Although a high variability of OBP gene expression is evident, our robust proteomic data clearly revealed that OBP99b, 19d, and 69a were the most abundant in female antennas. The latter provides more accurate information considering probable transcriptional, translational and post-translational regulation during odor perception. (Figure 3D, Table S3).

#### 2.3.2. Signal Transduction

The downstream signal transduction after odorant perception by receptors comprises a completely unexplored molecular area in tephritids. Our comparative proteomic approach also provides some clues on proteins associated with signal transduction of olfactory behavior. In this sense, we observed more upregulated proteins associated with signaling in the antennas of newly emerged flies exposed to CeraTrap^®^ over 24 h (24h_0D, Table 2) when compared with any of the other treatments. In the 24 h treatment, from a total of 35 proteins identified, 25 were upregulated in females and 29 in males and 22 proteins in both (Table 2).

The reticulon-like protein (Rtn1, Q9VMV9) was the only upregulated protein in the antennas of both newly emerged males and females exposed over two hours to CeraTrap^®^ (2h_0D, Table 2). Transient-receptor-potential-cation-channel-protein “painless” (Pain, Q9W0Y6), ubiquitin-40S ribosomal protein S27a (RpS27A, P15357), Calr, Ezrin-moesin-radixin 1 (EMR1, P46150), and Snmp1, were specifically upregulated in similarly aged females also treated over two hours with CeraTrap^®^ (Table 2). In contrast, cAMP-dependent, regulatory subunit type 2 (Pka-R2, P81900), reticulon-like protein (Rtn1_c, Q7KTP4), receptor activated protein kinase C 1(PKC, M9PCC1), Cam, sodium/potassium-transporting ATPase subunit alpha (Atpalpha, P13607), and voltage-dependent anion-selective channel (Porin, Q94920), were principally upregulated in newly emerged male flies (0 day old treatment) and in the case of Atpalpha (P13607) in five-day-old females and males exposed to CeraTrap^®^ over two and 24 h periods. By extending exposure time to CeraTrap^®^ to 24 h, also in newly emerged flies, antennas exhibited the upregulation of more proteins in both, males, and females (Table 2). Among these proteins the following were identified: RpS27A, Pain, Calr, Atpalpha, protein lap4 (LAP4, Q7KRY7), G protein alpha s subunit (P20354), porin, PRL-1 (Q95VY8), EMR1, sodium chloride cotransporter 69 (Ncc69, Q9VTW8), PKC, phosphatase 2A at 29B (Pp2A-29B, P36179), NADPH: adrenodoxin oxidoreductase (Dare, Q9V3T9), SPoCk, Rtn1, reticulon-like protein (Rtn1_b, E1JHT6), and Orco. Only in females did we observe the specific upregulation of Snmp1, aquaporin (Drip, Q9V5Z7), protein couch potato (Cpo, Q01617), calcium/calmodulin-dependent protein kinase type II alpha chain CaMKII, PKC, Pka-R2, and UDP-glucose 6-dehydrogenase (O02373). Males exhibited the exclusive upregulation of Rtn1_c, juvenile hormone-inducible protein 26 (Q7K0P0), innexin 2 (Inx2, Q9V427), plasma membrane calcium ATPase (PMCA, Q59DP9), scribbled, isoform S (Scrib, A0A0B4KHN3), and C-terminal binding protein (CtBP, A4V2S3).

Five-day-old flies exposed over two hours to CeraTrap^®^ (2h_5D, Table 2) also exhibited the simultaneous upregulation of RpS27A, Scrib, and Rtn1_c. However, mature males exhibited the upregulation of more proteins associated with signaling than females, including proteins such as G protein beta-subunit 13F (P26308), SPoCk, PKC, atpalpha, EMR1, porin, Ncc69, PRL-1, PMCA, Inx2, AMP deaminase (AMPdeam, Q9VY76), Calr, Cpo, Snmp1, Pain, CaMKII, and Pp2A-29B. Five-day-old flies exposed over 24 h to CeraTrap^®^ (24h_5D) and sexually mature flies (2/24_15D) did not exhibit any prominent upregulation of proteins associated with signaling.

#### 2.3.3. Odorant Degrading Enzymes (ODEs)

**Figure 4:**
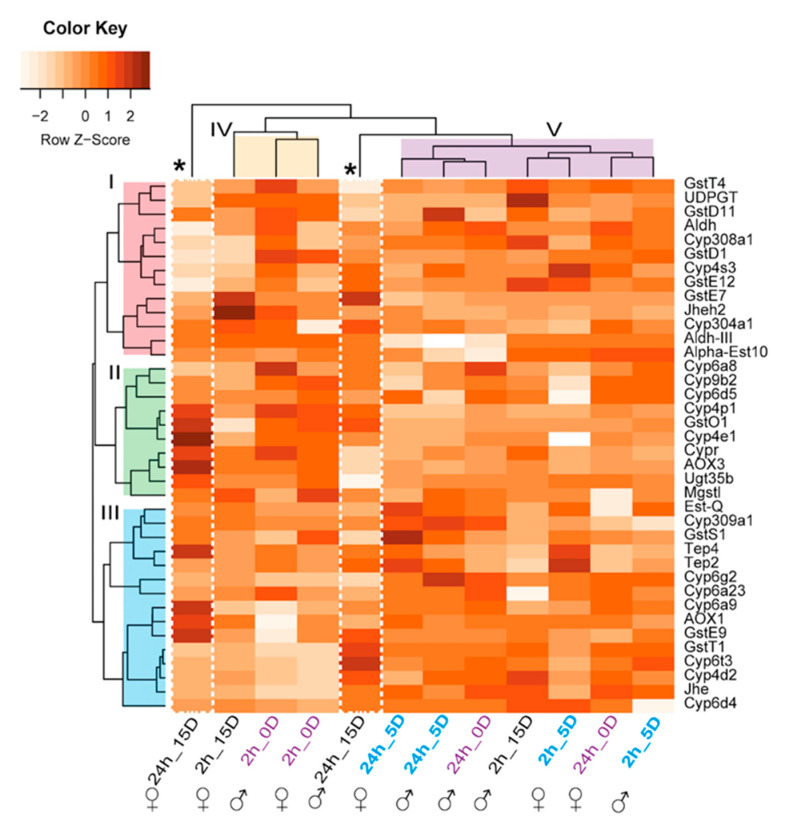
Odorant degrading enzymes upregulated in antennas of *A. ludens*, grouped according to treatments with CeraTrap®. Our proteomics study included antennas from newly emerged flies (2/24h_OD), and from five- (sexually immature; (2/24h_5D) and 15-day-old (2/24h_15D), sexually mature flies. Protein name abbreviation is indicated on the right side of heat map. Roman number on the left indicates clusters associated with protein ID and roman numbers at the top specify groups base protein relative abundance ratios (fold change: CeraTrap® / water). White dash rectangles and asterisk denote particularity in mature flies (24h_15D), where several ODEs displayed significant upregulation.

After signal perception, the content of odorant molecules must be regulated using a biochemical strategy. The degradation of odorants by odorant degrading enzymes (ODEs) could provide a plausible system. Consistent with this, our comparative proteomic approach allowed the identification of several proposed ODEs (Figure 4). The respective heatmap exhibited three main hierarchical clusters based on protein annotation (I–II) and two hierarchical groups based on relative protein abundance ratios (fold change: CeraTrap^®^/water, V and VI, Figure 4). Mature female and male flies exposed over 24 h to CeraTrap^®^ (24h_15D) exhibited a distinctive pattern of upregulation (indicated with an asterisk) compared to other treatments (Figure 4). In females, the following proteins associated to clusters II and III were highly upregulated: cytochrome Cyp4p1 (Q9V558), Cyp4e1 (Q9V4T5), Cypr (Q8IPJ7), GstO1 (Q9VSL6), AOX3 (Q9VF51), and UDP-glucuronosyltransferase (Ugt35b, Q9XYN3). Furthermore, the Cyp6a9 (Q27594), thioester-containing protein 4 (Tep4, M9PD73), AOX1 (Q9VF53), and GstE9 (Q7K8X7), in the group III, were also upregulated in sexually mature females (24h_15D).

Mature male flies treated with CeraTrap^®^ for 24 h (24h_15D) exhibited significant upregulation of GstE7 (Q8MR33), and the probable Cyp304a1 (Q9VG17) from cluster I, Cyp4p and GstO1 from cluster II, and GstE9, GstT1 (Q7K0B6), the probable Cyp6t3 (Q9V676), add Cyp4d2 (Q27589) all included in cluster II.

Immature flies (0D and 5D) did not exhibit significant upregulation in ODEs as was the case in sexually mature flies (15-D). Notably, antennas of newly emerged immature male flies (2/24_0D) exhibited more upregulated ODEs than females. Finally, we did not observe drastic upregulation of ODEs in five-day-old male and female flies exposed to CeraTrap^®^ (Figure 4).

## 3. Discussion

Our microscopy and nano-LC-MS/MS analyses comparing antennas stemming from female and male flies exposed to the proteinaceous attractant CeraTrap^®^ at various fly ages, yielded key insights on how the antennas of *A*. *ludens* respond to a potent attractant and allowed us to identify a core proteome featuring key proteins associated with odorant perception in this highly pestiferous fruit fly. As described before, we identified dense agglomerations of olfactory sensory neurons (OSNs) throughout the internal space of antennas stemming from newly emerged flies (Figure 1G), whereas in antennas from five (Figure 1H) and 15-day-old flies (Figure 1I) we identified a clear internal cavity surrounded by OSNs. In addition, our microscopy studies identified—in newly emerged flies—a notorious cuticle deposition in the external surface (“C” in Figure 1G) when compared to five and 15-day-old flies (Figure 1I). Furthermore, comparative proteomics studies of flies exposed to CeraTrap^®^ flushed out candidate molecular players related to the perception of this effective commercial lure. Our study provides the first global picture of the olfactory machinery of *A. ludens* at the protein level. We hope that in the mid to long term, our proteomics approach, coupled with transcriptomics, metabolomics, electrophysiological and behavioral studies, will pave the way to a full understanding of the molecular and behavioral bases of olfactory perception in this and other pestiferous species within *Anastrepha* and in other genera such as *Rhagoletis*, all being strongly attracted to protein derived lures (particularly the walnut infesting flies, *Rhagoletis* z*oqui* Bush and *Rhagoletis completa* Cresson and allow us to design new strategies for the biorational management of these economically important quarantine pests.

### 3.1. Flushing Out the Secrets of Olfactory Perception in A. ludens via the Characterization of the Antennal Proteome

Herbivores employ several strategies to carry out basic physiological tasks such as feeding, mating, oviposition, reproduction and repellency to molecules associated with predators and pathogens [37,41]. *Anastrepha ludens* is among the most pestiferous fruit fly species affecting the production of various tropical and subtropical fruits [17]. Several behavioral studies have provided a framework for the establishment of multiple strategies to overcome the negative effect of this pest [18,19,42]. However, to the best of our knowledge, there is very limited information on the molecular foundation of the most basic senses in tephritid flies, the olfaction. Several genomics studies have laid down the first set of sequences to start digging deeper into the olfaction molecular architecture of tephritid flies. In most of the cases, odor perception is a prerequisite to survival. Insects have the capacity to translate external signals into a behavioral responses following the perception of odors, which is carried out by the peripheral olfactory receptor neurons (ORNs), biochemical processing of the signal at the antennal lobes, and integration of signal transduction associated with olfaction in the higher processing center of the brain [5,43]. In general, volatile molecules penetrate the pore tubules of the sensillum and cross the hydrophilic sensillum lymph prior to reaching specific sensory dendrites, which triggers the signal transduction cascade leading to a behavioral response [44]. However, the vital complementation of genomics studies with other omics approaches such as proteomics and metabolomics will provide the next level of understanding/information for the establishment of the next generation of highly effective pest control management systems. Here, we provide the first glance into the proteome of *A. ludens* female and male antenna considering three maturity/physiological stages of adult development. In addition, our studies identify the occurrence of similar protein players in the olfactory system between *A. ludens* and *D. melanogaster*. It is noteworthy that the existence of similar protein players does not imply the same regulatory mechanism, which in most of the cases is controlled by the need to survive, find adequate food sources, and successfully compete and cope with a complex and variable external environment (epigenetic control) [45,46]. Our proteomics approach entailed using first in-solution and in-gel base trypsin digestion and nano-LC-MS/MS analyses in three different stages of antenna maturation, and then, a comparative proteomics study by carrying out a tandem mass tags (TMT) analysis combined with SPS-MS^3^ in *A. ludens* flies exposed to CeraTrap^®^ over short and long periods of time. In the front line, we could visualize several OBPs across different stages of antenna maturation (Table 1). Besides, proteins associated with calcium signaling and ODE were included as putative players of the regulation of CeraTrap^®^ perception in *A*. *ludens* antennas. This is a solid first step in our quest to understand the proteome related to the antennas of *A*. *ludens*. However, we still need to study important posttranslational modifications such as the glycosylation and phosphorylation of membrane and extracellular proteins, which could explain the lack of odorant receptors identified in our proteomics studies. We are aware that the very low level of expression that translates into the discovery of few abundant proteins, the particular neuronal location, and the exclusive sequences of odorant receptors, increase the complexity of profiling the complete proteome of the antenna of *A. ludens* and other flies [47]. However, combining multiple formats of affinity chromatography, mass spectrometry and a solid foundation of genomic information, will help us to further characterize the antenna proteome of *A. ludens* and other tephritid flies in the near future.

### 3.2. CeraTrap^®^ an Efficient Lure that Induces the Upregulation of the Olfactory Machinery of A. ludens

CeraTrap^®^ is an attractant formulated with enzymatically hydrolyzed protein obtained from pig intestinal mucosa during an unknown biopharmaceutical process to obtain heparin, which has exhibited much higher efficiency in trapping various species of *Anastrepha* when compared to other commercial protein-based baits [38,39,40]. Before dwelling on our comparative proteomics data, we would like to remind the reader that the effectiveness of proteinaceous attractants is closely associated with several physiological factors such as nutritional status, degree of sexual development, mating status, age, and gender [32,48]. Another important factor that needs to be kept in mind, is that protein represents a limiting resource in nature that provides essential amino acids to females for reproduction and to males for mating [49]. Therefore, efficiently finding protein sources in nature is of paramount importance for individuals as their fitness is closely related to this nutrient. Our data clearly show that more proteins associated with olfactory behavior (GO:0042048) were upregulated in antennas of immature flies (newly emerged and five-day-old) than in sexually mature flies (15-day old). Previous studies have suggested that sexually immature, protein-deprived flies were more attracted to CeraTrap^®^ [50], which support other studies indicating that immature, particularly female flies were more attracted to protein-based baits than mature and male flies due to protein requirements for future reproduction, as ovary development and egg production is intimately associated to protein ingestion [33,51,52]. However, females that were only fed on CeraTrap^®^ did not exhibit increased egg production [50], implying that other nutrients are necessary. Here, several OBPs exhibited more prominent upregulation in females exposed to CeraTrap^®^ over two and 24 h. Similarly, significant upregulation of OBPs was observed in five-day-old females exposed to with CeraTrap^®^ over two and 24 h. Besides, in most of the cases the amount of mRNA was corelated with proteomics data in the two-hour exposure treatment (Figure 3C,D). Our data therefore nicely agree with previous studies indicating that sexually immature females search for protein as it is essential for ovary/egg maturation and posterior mating and oviposition [31,51,53]. However, we should also consider the upregulation of OBPs in male flies. In fact, prominent gene expression of the OPB19d, 99c, 6d was observed in males exposed to CeraTrap^®^ over a two-hour period (Figure 3C,D). As is the case with females, immature males also need to ingest protein to guarantee reproductive success, which implies courtship, signaling, mating, and insemination [54,55,56]. In addition, protein is critical in the case of males to increase sperm load, which could be linked to the upregulation of some of the above-mentioned proteins [57]. Interestingly, males fed on CeraTrap^®^ obtained fewer matings than males fed on sugar, confirming that this attractant does not represent an adequate protein source for reproduction [50] or alternatively, may contain some additives in the formulation (mainly preservatives to reduce degradation and improve durability) that can negatively interact with the behavior and/or physiology of flies.

Our proteomics data indicate that OBP19d and OBP99a were upregulated in the antennas of newly emerged *A. ludens* adults and in five-day-old flies exposed to CeraTrap^®^ over two and 24 h. In addition, gene expression patterns in male and female antennas exhibited a negative direction in the same OPB19a and OBP99a when related to protein abundance (Figure 3C,D). These contrasting values might suggest a particular translational regulation that could be associated with the stability or compartmentalization of mRNA or delay in translation. In *Drosophila* both proteins have been annotated as secreted into the extracellular space (GO:0005615) and associated with the biological process of sensory perception of odors (GO:0007608). Gene expression of OBP19d has been observed on the anterior surface of the third antennal segment in *D. melanogaster* [58]. Additionally, gene expression of OBP99a has been linked in a subset of chemosensory sensilla located in the third larval segment of *D. melanogaster* were chemosensory sensilla are purportedly found [59,60]. In antennas of 15-day-old flies exposed to CeraTrap^®^ over two and 24 h, we observed a very limited upregulation of OBPs. Importantly, flies exposed over 24 h to CeraTrap^®^ exhibited the highest upregulation of ODEs, a fact possibly related to the limited response of mature flies to CeraTrap^®^. Among ODEs, Cyp6a9 has been associated with insect growth hormone metabolism as well as the breakdown of synthetic insecticides [61]. Besides, GstD1 identified in our study has displayed 1-chloro-4-[2,2,2-trichloro-1-(4-chlorophenyl)ethyl]benzene (DDT) dehydrochlorinase activity that may suggest its association with detoxification of the insecticide DDT [62]. Other ODEs upregulated in our proteomics study included the Tep4 annotated as extracellular protein (GO:0005615) and the Jheh2 annotated with the biological process of aromatic compound catabolic process (GO:0019439) and associated with the hydrolysis of the juvenile hormone (Unirule, PIRNR: PIRNR001112). However, most of these putative ODEs should be further tested to determine which active compound of CeraTrap^®^ is being metabolized in *A. ludens*.

**Figure 5:**
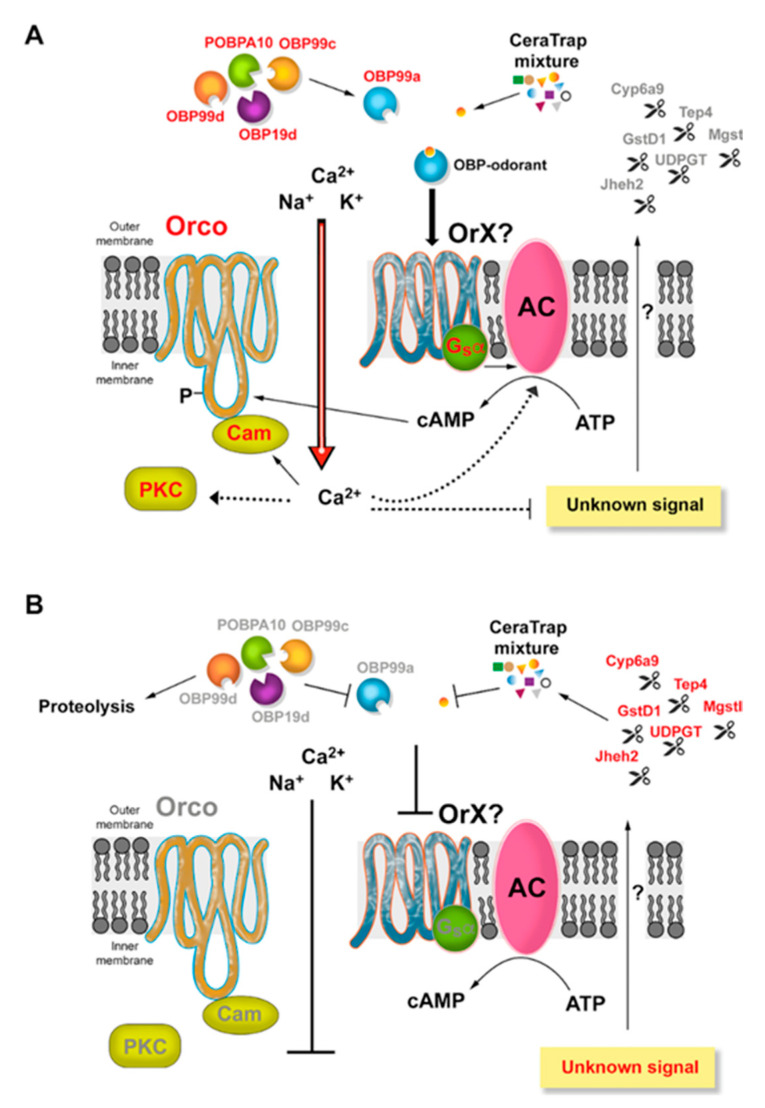
Model proposed for CeraTrap^®^ perception in *A. ludens* antennas based on antenna proteomics data. (**A**), Immature flies highly responsive to CeraTrap^®^ probably due to the upregulation of essential OBPs, an unknown receptor that in conjunction with Orco creates an influx of Ca^2+^ into the cell inducing the signal transduction of odor perception preferentially in female *A. ludens* flies. (**B**), In contrast, sexually mature flies are less responsive probably because of the upregulation of odorant degrading enzymes (ODE), which are activated through and known signal pathway. Proteins upregulated by CeraTrap^®^ are colored in red while proteins that did not show any change are depicted in grey. Dotted lines indicate a suggested connection, but further experimental validation is needed to validate this. Arrows indicate positive activation whereas flat lines point out inhibition.

The signal transduction pathway is the middle point between odorant perception and odorant catabolism. Therefore, a fine-tuned regulation must exist during odorant perception in *A. ludens*. Our proteomics data provide a valuable insight into the complexity of signaling in *A. ludens* under exposure to CeraTrap^®^. Besides, we are now able to corroborate some generalities suggested in previous studies (Figure 5). For example, under odorant perception, Orco was slightly upregulated in females in the two and 24 h treatments with CeraTrap^®^. This co-receptor forms a complex OrX/Orco creating a Ca^2+^ conducting cation channel activated by intracellular cAMP or cGMP [63,64], whose content is to some degree modulated by the upregulation of AMPdeam. Interestingly, our data showed that Snmp1 was over accumulated in CeraTrap^®^ treatments, which in concert with Orco, Or67d and OBP Lush are associated with detection and signal transduction of the fatty-acid-derived male pheromone 11-cis vaccenyl acetate (cVA) in *D. melanogaster* [63,65,66]. Besides, Cam and the PKC were slightly upregulated in female flies treated with CeraTrap^®^ over two and 24 h. These proteins modulate insect odorant receptors, possibly by affecting OR sensitization having as main target Orco due to the occurrence of conserved putative calmodulin (CaM)-binding motif [67] and by PKC phosphorylation [68]. This positive feedback could increase the intracellular levels of Ca^2+^, which induces proteins such as CaMKII and PMCA. Both proteins were upregulated in female flies treated with CeraTrap^®^ over two and 24 h. CaMKII is an essential regulator of plasticity in synaptic physiology and behavior, which is directly associated with the modulation of voltage-gated potassium channel through phosphorylation in *D. melanogaster* [69,70]. Under higher production of Ca^2+^, PMCA catalyzes the hydrolysis of ATP coupled with the transport of calcium out of the cells providing homeostasis of cellular Ca^2+^ (GO:0006874, cellular calcium ion homeostasis).

### 3.3. Integration of Proteomic Data to Help the Establishment of a New Generation of Tools for the Biorational Management of A. ludens

CeraTrap^®^ purportedly consists of a complex mix of yet undetermined peptides, proteins, and metabolites from pig intestinal mucosa. Based on current information, we suggest that volatile compounds from the protein content of CeraTrap^®^ could be the main attractant, but the question remains as to which volatile or volatiles emanating from the protein/peptides forming part of the attractant generate the powerful response in flies to CeraTrap^®^. By tracking the upregulation of OBPs and ODEs during behavioral studies, we could possibly get closer to unravelling this perfect mix. In fact, the common upregulation of OBP19d and OBP99a in newly emerged and five-day-old antennas of flies exposed to CeraTrap^®^ over two and 24 h, could provide an invaluable biochemical resource that can make the purification of active compounds in CeraTrap^®^ possible, a task we are currently involved in. Furthermore, the common over accumulation of OBP19a in antennas of newly emerged flies, and mutual upregulation of OBP99c_B and POBPA10 in antennas of five-day-old flies (Figure 2B), could suggest that antennas in differently-aged flies use specific sets of OBPs for the perception of CeraTrap^®^ active molecules. In addition, more in-depth analyses of the upregulation of ODEs such as GstD1, Tep4, Jheh2, and Ugt35b in *A. ludens* exposed to CeraTrap^®^, could help in understanding the mechanism through which these enzymes break down the active molecules of this attractant.

CeraTrap^®^ elicits a strong behavioral response by wild flies in field tests [38,39,40] and in line with this, our proteomics data strongly suggest the upregulation of the olfactory system of *A. ludens*, which encourages us to further scrutinize this potent attractant. In this context, the proteomics tool can provide more valuable insights than conventional antennal electrophysiological tests, which mostly fail to deliver positive recordings by themselves [71,72,73]. For example, the main apple volatiles induce strong reactiveness in the antenna of codling moths, but this does not correlate with any behavioral response [74,75,76]. Before conducting any further molecular and biochemical studies, we should corroborate the association of OBP19d and OBP99a in immature flies with perception of CeraTrap^®^. Further molecular characterization with CRISPR-Cas9 gene editing technology or RNAi technology to knockout or induce the overexpression via other molecular tools, might underpin the role of OBP19d and OBP99a in the perception of CeraTrap^®^ active molecules for *A. ludens*. Finally, protein–protein interaction tools could provide a complete repertoire of molecules interacting with OBP19d and OBP99a, including target odorant receptors, in which deorphanization could be corroborated by other approaches such as the heterologous mutant expression system (the “empty-neuron”) of *Drosophila* [73,77,78].

## 4. Materials and Methods

### 4.1. Chemical Reagents

Reagents used were purchased from Sigma-Aldrich (St. Louis, MO, USA) or from other companies specified in the corresponding section.

### 4.2. Insect Rearing and Study Site

*Anastrepha ludens* flies were obtained from a colony established in the Red de Manejo Biorracional de Plagas y Vectores at the BioMimic^®^ Scientific and Technological Cluster of the Instituto de Ecología, A.C. (INECOL), in Coatepec, Veracruz, Mexico. Flies were reared for 150 generations (refreshed with wild material in March/2013) following methods described in [19]. After emergence from pupae, adults were separated by sex, kept in plexiglass cages (30 × 30 × 30 cm) and fed *ad libitum* with hydrolyzed protein and sugar (1:3 ratio) and water. The rearing of insects and assays were carried out at 27 ± 1 °C, 70 ± 5% relative humidity and 12:12 h L/D photoperiod. Proteomics analyses were performed in the proteomics laboratory of the Red de Estudios Moleculares Avanzados at the BioMimic^®^ Cluster.

### 4.3. Light Microscopy (LM) Studies

Antennas were removed from newly emerged (0D), five-day-old (5D) and 15-day-old (15D) fruit flies (females) in a laboratory kept in the same environmental conditions as those used for attractant exposures with the help of fine-point, stainless steel watchmaker tweezers. A total of five antennas per treatment were collected (overall total of 15 antennas). After dissection/removal from the head, antennas were fixed for 8 h in 4% paraformaldehyde and 5% glutaraldehyde in 0.1 M sodium cacodylate at pH 7.2, rinsed overnight in the same buffer, post-fixed in 1% OsO4 at 4 °C for 1 h, and then dehydrated using gradually increasing ethanol concentrations (30–100%) during 10 min at each concentration. Samples were then embedded in LR-White resin (Sigma-Aldrich) and polymerized at 55 °C overnight. Then, 1 µm thick transverse sections of the antennas were obtained with the aid of an ultramicrotome EMUC7 (Leica Microsystems GmbH, Wien, Austria). The sections were stained with toluidine blue and images were obtained with a Leica DMI6000B microscope (Leica Microsystems CMS GmbH, Mannheim, Germany) with LAS AF software (v.4.0.0.11706), using an apochromatic plan 63× (NA 1.4, oil) objective.

### 4.4. Confocal Scanning Laser Microscopy (CSLM)

Samples were observed immediately after dissection. The images were obtained with a TCS-SP8+STED microscope (Leica Microsystems CMS GmbH, Mannheim, Germany) using HC-PL Fluotar 20× (NA 0.50, dry) objective. The chitin autofluorescence of samples were recorded in a yellow channel at 586–671 nm emission; excitation 488 nm.

### 4.5. Scanning Electron Microscopy (SEM)

Samples were fixed in 2.5% glutaraldehyde buffered with Sorenson’s phosphate for 12 h at 4 °C, rinsed twice in the same buffer for five minutes. The tissues were then dehydrated in a graded ethanol series (30–100%) for 30 min at each concentration, dried in a Quorum K850 critical point drying with CO_2_ and attached to aluminum stubs using a carbon adhesive before coating with gold in a sputtering Quorum Q150 RS [79]. The preparations were studied and photographed with an FEI Quanta 250 field emission gun (FEG)scanning electron microscope (FEI Inc., OR, USA).

### 4.6. Anastrepha ludens Exposure to CeraTrap^®^

Previous assays in laboratory and field cages have documented the high effectiveness of the proteinaceous attractant CeraTrap^®^ (Batch 14/0090, Bioiberica, Barcelona, Spain) in attracting laboratory-reared *A. ludens* flies, in the same order of magnitude as wild flies [38]. Based on the latter, and to guarantee simultaneous access to homogeneous material in the large numbers needed, we felt justified to use laboratory flies for this assay. Prior to exposure to the attractant, pupae were maintained at 22 ± 1 °C, 70 ± 5% relative humidity and dark conditions, then transferred for adult emergence to 30 × 30 × 30 cm cages placed in a clean room without traces of any fruit or protein-based volatile under controlled conditions (27 ± 1 °C, 70 ± 5% relative humidity and 12:12 h L/D photoperiod). After emergence from puparia, batches of 150 flies, females and males, were separately selected based on age: (i) newly emerged (0D) 0–6 h; (ii) five-day-old flies (5D) considered sexually immature based on gonad development [31]; and (iii) 15 day-old flies (15D) considered sexually mature. Antennas of flies exposed over periods of two and 24 h to water or CeraTrap^®^ were analyzed. Exposure to volatiles was completed using 50 mL of CeraTrap^®^ or distilled water placed in a container (10.8 cm in diameter × 4.2 cm in height) with a perforated lid and equipped with a mesh that allowed the exchange of odors, but prevented the passage of the flies to the attractant. To avoid odor contamination, flies were exposed in two independent rearing rooms that had been thoroughly cleaned to remove any possible sources of extraneous volatiles.

Antennas from one hundred male and female flies (total of 200) were collected by carefully removing individual antenna from the fly’s heads with the help of an entomological pin and watchmaker tweezers (Dumont style 5), pooled in 50 µL of phosphate buffered saline (PBS), pH 7.4 (Cat. P5493-1L, Sigma-Aldrich) plus sodium dodecyl sulfate (SDS) 4% and stored at –80 °C until the proteomic analysis was run. All experiments were carried out per triplicate.

### 4.7. Protein Extraction

The pool of antennas in PBS from each treatment was grounded with a sterile pistil inside of the tube (1.5 mL) for three minutes on ice. Thereafter, the mixture was sonicated for five minutes, boiled for five minutes and centrifuged at 10620 ×*g* for five minutes. Afterwards, the supernatant was recovered and stored at –80 °C for further analysis. The protein assay was carried out using the bicinchoninic acid (BCA)™ Kit Assay (Cat. No. 23225, Pierce; Rockford IL, USA) using bovine serum albumin as standard. In addition, the quality of the protein extract was corroborated by fractionating 10 µg of total protein on an any kD^TM^ Tris-glycine SDS-PAGE gel (Cat. 4569033, TGX™ gels, BioRad; Hercules, CA, USA) and stained with SYPRO Ruby protein gel stain (Cat. S12001, Life Technologies, Grand Island, NY, USA).

### 4.8. Trypsin Digestion, TMT Labeling, Simple Fractionation and Desalting

In-gel and in solution digestions were analyzed as previously reported [80,81]. Briefly, reduced and alkylated proteins from SDS-PAGE and protein crude extracts (100 µg) were digested with trypsin (Trypsin Gold, Mass Spectrometry Grade, Promega, Madison WI, USA) at a 1:30 *w*/*w* trypsin protein ratio overnight at 37 °C. Thereafter, a 1:60 *w/w* freshly prepared trypsin solution was added during 4 h at 37 °C. Samples for analysis via nano-LC-MS/MS were fractionated using strong cation exchange (SCX) cartridges (Thermo Scientific, Bellefonte, PA, USA) and desalted with C18 cartridges and dried in a CentriVap vacuum concentrator (Labconco, Kansas, Missouri, USA). In addition, peptide samples for comparative proteomics were labeled with TMT 6-plex reagents just after tryptic digestion, according to manufacturer’s instructions (Thermo Fisher Scientific, Rockford, IL, USA). The labels 126, 127 and 128 were used for control treatments consisting in water, while 129, 130, and 131 tags were used for the CeraTrap^®^ treatment. Labeled samples were pooled for further processing.

### 4.9. Fractionation of Labeled Peptide Mixture

Pooled TMT-labeled samples were fractionated using strong cation exchange (SCX) cartridges (Thermo Scientific, Bellefonte, PA, USA) and following the instructions of the manufacturer. Five fractions were collected: fluently, 75, 250, and 500 mM KCl. Each fraction was dried using a CentriVap vacuum concentrator (Labconco, Kansas, Missouri, USA) and desalted with C18 cartridges for MS analysis according to manufacturer’s guide.

### 4.10. Nano-LC-MS/MS Analysis

Samples were analyzed by nano-LC-MS/MS using an Orbitrap Fusion Tribid (Thermo-Fisher Scientific, San Jose, CA) mass spectrometer equipped with an “EASY spray” nano ion source (Thermo-Fisher Scientific, San Jose, CA, USA). The samples were reconstituted with 0.1% formic acid in LC-MS grade water (Solvent A) and 20 μL was injected into a nanoviper C18 trap column (3 µm, 75 µm × 2 cm, Dionex) at 3 μL/min flow rate in an UltiMate 3000 RSLC system (Dionex, Sunnyvale, CA). After this procedure, it was separated on an EASY spray C-18 RSLC column (2 µm, 75 µm × 25 cm), using a 100 min gradient with a flow rate of 300 nL/min, and using solvent A together with 0.1% formic acid in 90% acetonitrile (Solvent B). The gradient was as follows: 10 min solvent A, 7–20% solvent B within 25 min, 20% solvent B for 15 min, 20–25% solvent B for 15 min, 25–95 % solvent B for 20 min, and 8 min solvent A. The mass spectrometer was operated in positive ion mode with nanospray voltage set at 3.5 kV and source temperature at 280 °C. External calibrants included caffeine, Met-Arg-Phe-Ala (MRFA) and Ultramark^®^ 1621 (88323, Thermo Fisher Scientific^TM^ Pierce^TM^).

### 4.11. Decision Tree-Driven MS/MS

The mass spectrometer was operated in a data-dependent mode. Briefly, survey full-scan MS spectra were acquired in the Orbitrap analyzer, scanning of mass range was set to 350–1500 m/z at a resolution of 120,000 (FWHM) using an automatic gain control (AGC) setting to 4.0e5 ions, maximum injection time to 50 ms, dynamic exclusion 1 at 90S and 10 ppm mass tolerance. Subsequently, a top speed survey scan for 3 s was selected for subsequent decision tree-based Orbitrap collision induced dissociation (CID) or higher-energy collisional dissociation (HCD) fragmentation [82,83]. The signal threshold for triggering an MS/MS event was set to 1.0e4 and the normalized collision energy was set to 35 and 30% for CID and HCD, respectively. The AGC of 3.0e4 and isolation window of 1.6 m/z was set for both fragmentations. Additional parameter for CID included activation Q was set to 0.25 ms and injection time to 50 ms. For HCD, first mass was set to 120 m/z and injection time to 100 ms. The settings for the decision tree were as follows: for HCD fragmentation charge states two or three were scanned in a range of 650–1200 m/z, charge states four were scanned in a range of 900–1200 m/z, and charge states five were scanned in a range of 950–1200 m/z. For CID fragmentation charge states three were scanned in a range of 650–1200 m/z, charge state four were scanned in a range of 300–900 m/z, and charge state five were scanned in a range of 300–950 m/z. All data were acquired with the Xcalibur^TM^ 4.0.27.10 software (Thermo-Fisher Scientific).

### 4.12. Synchronous Precursor Selection (SPS)-MS3 for TMT Analysis

Full MS scans were run in the Orbitrap analyzer with a 120,000 (FWHM) resolution, scan range 350–1500 m/z, AGC of 2.0e5, maximum injection time of 50 ms, intensity threshold of 5.0e3, dynamic exclusion one at 70s and 10 ppm mass tolerance. For MS2 analysis, the 20 most-abundant MS1s were isolated with charge states set to 2–7. Fragmentation parameters included, CID with 35% of collision energy and activation Q of 0.25, AGC of 1.0e4 in maximum injection time of 50 ms, precursor selection mass range of 400–1200 m/z, precursor ion exclusion width low 18 m/z and high five m/z, isobaric tag loss TMT and detection run in the ion trap. Afterwards, MS3 spectra were acquired as previously described [84] using synchronous precursor selection (SPS) of 10 isolation notches. MS3 precursors were fragmented by HCD with 65% of collision energy and analyzed using the Orbitrap with 60,000 resolution power at 120–500 m/z scan range, two m/z isolation window, 1.0e5 AGC and maximum injection time of 120 ms with one microscan.

### 4.13. Data Analyses and Interpretation

The spectra were processed with Proteome Discoverer^TM^ 2.1 software (PD, Thermo Fisher Scientific Inc.) and searches conducted with Mascot 2.4.1 software (Matrix Science Inc., Boston, MA, USA), SQUEST HT [85], and MS AMANDA database search algorithm engines [86]. The searches were directed against a translated unigene database generated with in-house transcriptomic data from *A. ludens*. Parameters in the search included: full-tryptic protease specificity, two missed cleavages allowed. In addition, static modifications covered carbamidomethylation of cysteine (+57.021 Da) and TMT 6-plex N-terminal/lysine residues (+229.163 Da). Dynamic modifications included methionine oxidation (+15.995 Da) and deamidation in asparagine/glutamine (+0.984 Da). For the synchronous precursor selection SPS-MS3 method, in which identification was performed at a lower resolution in the linear ion trap, tolerances of ± 10 ppm and ± 0.6 Da were applied. Resulting peptide hits were filtered for maximum 1% false discovery rate (FDR) using the percolator algorithm [87]. The TMT 6-plex quantification method within PD software was used to calculate the reported ratios applying mass tolerances of ±10 ppm in the case of the most confident centroid and a precursor co-isolation filter of 45%. For the SPS-MS3 method, the quantification was run at the MS3 level. Proteins were classified based on GO ontology enrichment of biological processes using *Drosophila* protein homologous proteins and the David ontology tool (https://david.ncifcrf.gov/) [88,89]. We used the REVIGO web server (http://revigo.irb.hr/) with a median similarity for the visual representation of the clustering of biological processes [90]. The mass spectrometry proteomics data have been deposited in the ProteomeXchange Consortium via the PRIDE [91] partner repository with the dataset identifiers PXD01996 and PXD020012.

### 4.14. Real Time-Quantitative Polymerase Chain Reaction (RT-qPCR) Analysis

Validation of proteomic data was performed by RT-qPCR using RNA samples of five-day-old *A*. *ludens* antennas. Separated female and male flies from laboratory were exposed to 50 mL of distilled water or CeraTrap^®^ over two hours, as previously described. Once flies were exposed, antennas were dissected and placed in 2 mL tubes kept on ice. Three biological replicates with samples consisting of 200 antenna pools were stored at -80 °C until RNA extraction. RNA was isolated using the RNeasy Plant Mini Kit (Qiagen, Hilden, Germany) following the manufacturer´s instructions. Integrity of total RNA was checked by agarose gel electrophoresis and ratio A260/A280 close to 2.0 measured in a BioSpec-nano spectrophotometer (Shimadzu, Kyoto, Japan). Following the protocols of the manufacturer, the total RNA was treated with DNAse I (Thermo Scientific, CA, USA), then cDNA was obtained from one microgram of total RNA using SuperScript^TM^ III Reverse Transcriptase (Invitrogen^TM^, CA, USA), and cDNA was finally treated with RNAse H (2U) (Invitrogen^TM^, CA, USA) to remove RNA traces and diluted to 10 ng/μL.

For qPCR, primers were designed based on protein sequences using Primer-BLAST program (National Center for Biotechnology Information-NCBI) for nine OBPs found upregulated in proteomic analysis (Table S3). Two genes were used as reference control: *A. ludens Tubulin* (UN025693) and *Actin* (UN031841) (Table S3). A dissociation curve was performed to evaluate the specific amplification of primers. The amplification efficiency of selected genes was higher than 95% with a correlation coefficient (R^2^) > 0.95.

RT-qPCR was performed in a STRATAGEN MX3005P QPCR System (Agilent Technologies, CA, USA) using Quantinova^TM^ SYBR Green RT-PCR Master Mix (Qiagen, Hilden, Germany). The amplification protocol consisted of: 95 °C for two minutes followed by 40 cycles of 95 °C for 5s, 60 °C for 15 s and 72 °C for 10 s. A melting curve was analyzed at 95 °C for 5 s, and increments of temperature from 60 °C for 15 s to 94 °C for 10 s. Three technical replicates and three biological samples were analyzed for each condition. The relative normalized expression of each OBP was calculated using the 2^–ΔΔCt^ method [92].

## 5. Conclusions

Our study, represents, to the best of our knowledge, the first comprehensive investigation on the proteomics of antennas of a tephritid fly exposed to the highly effective proteinaceous attractant CeraTrap^®^ shortly after adult emergence, five days thereafter, and at 15 days of age, when flies have fully reached sexual maturity. Altogether, CeraTrap^®^ performs as a potent attractant, possibly containing the perfect balance of proteins eliciting behavioral responses by fruit flies, thus representing an ideal attractant/tool for scrutinizing the molecular olfactory machinery of *A. ludens* and other economically important flies that so strongly respond to this bait [71]. The interesting results obtained here, which significantly expand the current knowledge in the field, encourage us to seek further insights into the molecular and behavioral basis of odor perception and processing in this biologically interesting, and at the same highly pestiferous group of insects, and to persist in our quest to produce an even more potent attractant than CeraTrap^®^. If successful, we could more accurately monitor adult populations and kill many females before they reach sexual maturity, mate and start laying eggs into valuable fruit, thus supporting environmentally friendly, biorational pest management schemes. On the other hand, we would be contributing to the broader goal of better understanding odor perception in insects by adding a defined ecological context to the studies.

## Figures and Tables

**Table 1:** Key examples of proteins associated with odorant perception featured in the core proteome of *A. ludens*’s antennas of different ages (antennas from newly emerged flies (0D), and from five- (sexually immature, 5D) and 15-day old, sexually mature flies, 15D) identified by nano-LC-MS/MS.

Name.	Search Engine Score			
	Coverage	Mascot	Sequest	Amanda
**Response to Pheromone (GO:0019236)**				
Odorant-binding protein 99b(Obp99b)	20.3	41.6	8.9	1005.1
Odorant-binding protein 19a(Obp19a)	5.4	60.4	2.2	329.1
Odorant-binding protein 99a(Obp99a)	20.2	289.5	50.3	3044.6
Odorant-binding protein 28a(Obp28a)	23.9	195.2	24.6	2393.9
General odorant-binding protein Lush (OBP Lush)	18.9	58.2	10.5	1392.3
Odorant-binding protein 19d(Obp19d)	8.8	261	22.4	873.6
Odorant-binding protein 99c, isoform B(Obp99c_B)	11.6	128.1	10.9	1270.6
Odorant-binding protein 83a(Obp83a)	42.9	460.1	59.4	2532.2
Odorant-binding protein 69a(Obp69a)	23.9	218	28.1	500.4
Odorant-binding protein 44a(Obp44a)	22.5	158.9	16.5	2334.1
Putative odorant-binding protein A10	51.6	586.3	87.2	7396
Sensory neuron membrane protein 1(Snmp1)	12.1	68.3	9.7	1222.2
Odorant receptor co-receptor (Orco)	13.5	58.2	7.9	948.7
**Cellular Calcium Ion Homeostasis (GO:0006874)**				
Cation-transporting ATPase	6.5	62.3	9.1	1085.3
Ryanodine receptor (RyR)	1.1	94.8	8.8	800.4
upheld(up)	32.2	564.1	105.7	10436
Plasma membrane calcium ATPase (PMCA)	14.3	349.3	68	3815.5
Inositol 1,4,5,-tris-phosphate receptor(Itp-r83A)	4.4	182.8	19.2	1969.8
Calreticulin (Calr)	18.1	185.7	44.2	4205.3
Calnexin 99A(Cnx99A)	25.2	269	49.2	5100.4
Calmodulin (Cam)	79.1	513.6	73.1	5233.8
Calcium/calmodulin-dependent protein kinase II(CaMKII)	18.8	193.5	22.4	2524
Secretory pathway calcium atpase (SPoCk)	8.5	238.2	30.9	1887
Calmodulin-binding protein related to a Rab3 GDP/GTP exchange protein (Crag)	2.9	56.6	3.2	551.3
Calcium-independent phospholipase A2 VIA (iPLA2-VIA)	8.3	116.2	18.1	1589.9
**Putative Odorant-Degrading Enzymes (ODE)**				
Cytochrome c oxidase subunit 5B(COX5B)	10.8	56.3	5.4	471.5
Cytochrome c oxidase subunit 5A(COX5A)	16	247	34	2052.4
Cytochrome c oxidase subunit 5A(COX5A)	16	247	34	2052.4
Cytochrome c oxidase subunit 7A(COX7A)	9.2	25	1.9	181.7
Glutathione S transferase E7(GstE7)	26.1	241.4	39.3	3324.3
Juvenile hormone epoxide hydrolase 2(Jheh2)	22.8	189.1	32.4	2632.6
Thioester-containing protein 4(Tep4)	16	502.5	68.1	6495.8
Cytochrome P450-4d2(Cyp4d2)	6.4	72.7	7.2	728.7
Cytochrome P450-9b2(Cyp9b2)	15.5	170.6	35	3672.4
Cytochrome P450 reductase (Cpr)	21.2	512.2	89	7761.4
Cytochrome P450-4e1(Cyp4e1)	27.7	230.8	40.6	3214.5
Cytochrome P450-6a8(Cyp6a8)	13.8	162.4	21.2	1942.7
Cytochrome P450 6a2	14.8	40.8	2.7	216.2
Probable cytochrome P450 6a23	7.6	119.3	11	1013.7
Glutathione S transferase S1(GstS1)	22.5	179.7	32.6	3020.1
Glutathione S transferase E9(GstE9)	19	281.4	30.7	3201.6
Glutathione S transferase D11(GstD11)	30.6	300.9	36.5	3926.5
Glutathione S-transferase D1	38.1	740.2	136.1	8744.6
Microsomal glutathione S-transferase-like (Mgstl)	8.4	54.22	3.3	398.9
Alpha-Est10	5.4	35.5	2.3	465.8
Thioester-containing protein 2(Tep2)	3.2	48	10.4	912.2
Thioester-containing protein 2(Tep2)	18.8	877.7	180	14738.1
Thioester-containing protein 3(Tep3)	5.4	86.9	22.1	1787.1
Aldehyde dehydrogenase (Aldh)	28.4	358.5	61.6	4366.4
Aldehyde oxidase 3(AOX3)	11.4	705.6	112.3	11329.5
UDP-glycosyltransferase 35b(Ugt35b)	5.8	25.5	1.9	325.1
Aldehyde oxidase 1 (AOX1)	6.8	236.5	37.3	2716

**Table 2:** Proteins associated with signal transduction of odor perception in antennas of *A. ludens* exposed over two or 24 h to the proteinaceous attractant CeraTrap^®^. Our proteomics studies included antennas of newly emerged flies (2/24h_0D), five- (sexually immature; 2/24h_5D) and 15-day old, sexually mature flies (2/24h_15D).

Proteins	Code	0D				5D				15D			
		2 h		24 h		2 h		24 h		2 h		24 h	
		F	M	F	M	F	M	F	M	F	M	F	M
		Abundance Ratio:(CeraTrap^®^)/(Water)											
Ubiquitin-40S ribosomal protein S27a (RpS27A)	P15357	**2.0**	1.4	**2.1**	**1.7**	**2.4**	**2.1**	0	1.2	0	1.1	**2.2**	1.2
Reticulon-like protein (Rtn1_c)	Q7KTP4	0	**1.5**	1.4	**1.7**	**1.6**	**1.7**	1.3	1.4	0	1.0	**1.9**	1.0
Sodium/potassium-transporting ATPase subunit alpha (Atpalpha)	P13607	0.9	**1.7**	**1.7**	**1.7**	**1.5**	**1.7**	**1.5**	**1.5**	0.3	1.0	**1.9**	1.1
PRL-1 phosphatase (PRL-1)	Q95VY8	0	0	**1.6**	**1.7**	1.3	**1.6**	1.3	**1.5**	0	0	0	1.3
Moesin/ezrin/radixin homolog 1, Ezrin-moesin-radixin 1 (EMR1)	P46150	**1.8**	1.4	**1.6**	**1.6**	**1.5**	**1.7**	1.4	1.4	0.6	1.3	**2.1**	1.1
Juvenile hormone-inducible protein 26	Q7K0P0	0.7	0	1.4	**1.6**	1.3	1.4	**1.5**	1.3	0	1.4	0	1.1
Innexin inx2 (Inx2)	Q9V427	1.4	0	**1.5**	**1.6**	1.3	**1.6**	1.3	1.4	0	1.1	0	0
Voltage-dependent anion-selective channel (Porin)	Q94920	1.0	**1.7**	**1.6**	**1.6**	**1.5**	**1.7**	1.4	**1.6**	0.1	1.0	**3.0**	1.1
G protein alpha s subunit	P20354	0	0	**1.6**	**1.6**	1.1	**1.5**	1.4	1.4	0	1.1	0	**1.6**
plasma membrane calcium ATPase (PMCA)	Q59DP9	0.7	1.3	1.4	**1.6**	1.3	**1.6**	**1.5**	1.3	1.2	1.2	0	1.0
Secretory Pathway Calcium atpase (SPoCk)	Q9VNR2	0	0	**1.5**	**1.6**	1.1	**1.7**	1.3	1.4	0	1.3	0	0
Scribbled, isoform S (Scrib)	A0A0B4KHN3	0	0	1.1	**1.6**	**1.8**	**1.7**	1.3	1.2	0	0	0	1.1
Protein lap4 (LAP4)	Q7KRY7	0	1.3	**1.7**	**1.6**	1.0	1.4	1.3	1.4	0	1.3	**2.3**	1.3
Transient receptor potential cation channel protein painless (Pain)	Q9W0Y6	**2.3**	1.4	**1.7**	**1.6**	1.3	**1.5**	1.3	1.4	0	1.2	0	1.0
C-terminal Binding Protein	A4V2S3	0.7	0	1.4	**1.6**	1.2	1.4	**1.5**	1.3	0	1.1	**2.7**	1.3
NADPH:adrenodoxin oxidoreductase (Dare)	Q9V3T9	0	0	**1.5**	**1.6**	1.2	1.4	1.4	1.3	0	1.1	0	1.1
Calreticulin (Calr)	P29413	**1.8**	1.3	**1.7**	**1.6**	1.4	**1.5**	**1.5**	1.2	0.6	1.4	0.8	1.1
Protein phosphatase 2A at 29B (Pp2A-29B)	P36179	0	**1.5**	**1.5**	**1.6**	1.0	**1.5**	1.1	1.2	0	1.1	0	1.4
Sodium chloride cotransporter 69 (Ncc69)	Q9VTW8	0.8	0	**1.6**	**1.5**	1.4	**1.7**	1.3	1.4	0	1.1	0	1.2
Reticulon-like protein (Rtn1)	Q9VMV9	**1.8**	**1.5**	**1.5**	**1.5**	**1.5**	1.4	1.4	1.3	0.3	1.1	0	1.0
Reticulon-like protein (Rtn1_b)	E1JHT6	0	0	**1.5**	**1.5**	**1.5**	1.4	1.4	1.3	0.1	1.1	0	1.0
Receptor of activated protein kinase C 1n (PKC)	M9PCC1	0.7	**1.5**	**1.5**	**1.5**	1.4	**1.7**	1.4	**1.6**	0	1.4	0	1.0
Protein couch potato (Cpo)	Q01617	0	0	**1.6**	**1.5**	1.4	1.5	1.3	1.4	0	**1.5**	0	1.0
Protein kinase, cAMP-dependent, catalytic subunit 1	P12370	0	0	0	**1.5**	1.2	1.4	1.3	1.4	0	1.0	0	1.2
Protein kinase, cAMP-dependent, regulatory subunit type 2	P81900	0	**1.6**	**1.6**	**1.5**	1.4	**1.5**	1.3	0	0	1.1	0	**1.7**
G protein beta-subunit 13F	P26308	0	1.2	1.3	**1.5**	1.4	**1.7**	1.3	0	0	1.2	0	1.0
Calcium/calmodulin-dependent protein kinase type II (CaMKII)	Q00168	0	0	**1.6**	**1.5**	1.2	**1.5**	**1.5**	1.4	0	1.1	0	0
Sensory neuron membrane protein 1 (Snmp1)	Q9VDD3	1.6	1.4	**1.8**	**1.5**	1.1	**1.5**	1.4	**1.5**	0.9	1.3	0	1.0
Calmodulin	P62152	1.0	**1.5**	**1.5**	1.4	1.2	**1.5**	1.4	1.2	0	**1.6**	0	1.1
AMP deaminase (AMPdeam)	Q9VY76	0	0	1.4	1.4	1.3	**1.6**	1.4	1.4	0.4	1.0	0	0
Adenylate kinase 3	Q9VGU6	0	0	1.3	1.4	1.2	1.3	1.2	1.3	0	1.0	0	1.1
UDP-glucose 6-dehydrogenase	O02373	0	0	**1.8**	0	0	0	0	0	0	1.0	0	0
Aquaporin	Q9V5Z7	0	0	**1.7**	0	1.1	0	1.3	1.4	0.1	1.0	0	0
Receptor-type guanylate cyclase	Q7JQ32	0	0	0	0	1.3	0	0	0	0.1	0	0	0
Odorant receptor co-receptor (Orco)	Q9VNB5	0.6	0	**1.5**	**1.5**	1.2	1.4	1.3	**1.6**	0	1.4	0	0

M: male, F: female. Abundance Ratios in bold indicate protein over accumulation in female and male flies.

## Supplementary Materials

Supplementary materials can be found at https://www.mdpi.com/1422-0067/21/21/8086/s1. Additional file 1: Table S1. Proteins identified by nano-LC-MS/MS analysis in each tissue. Gene ontology (GO) enrichment of the core proteome-based David functional annotation bioinformatics microarray analysis (https://david.ncifcrf.gov/) using Drosophila homologs. We used the REVIGO web server (http://revigo.irb.hr/) for GO clustering and visual representation of biological processes. Additional file 2: Table S2. Proteomic and mass spectrometric data of odorant-binding proteins identified by TMT-SPS-MS3 approach. The information includes peptide sequences, search engine score and abundances base TMT. Additional file 3: Table S3. Primers, qRT-PCR data and statistical analysis. Databases are available via ProteomeXchange with identifiers PXD019960 and PXD020012.

## Abbreviations

Ar	Arista
AOX	Aldehyde oxidases
BP	Biological processes
Calr	Calreticulin
Cam	Calmodulin
CaMKII	Calcium/calmodulin-dependent protein kinase II
COX	Cytochrome c oxidases
Cpo	protein couch potato
CSLM	Confocal scanning laser microscopy
CSP	Chemosensory proteins
Cyp DDT	Cytochromes P450 1-chloro-4-[2,2,2-trichloro-1-(4-chlorophenyl)ethyl]benzene
EMR	Ezrin-moesin-radixin
EST	Expressed sequence tag
Flag	Flagellums
GO	Gene ontology
GRs	Gustatory receptors
Gst	Glutathione *S* transferases
IPM	Integrated pest management
IRs	Ionotropic receptors
iTRAQ	Isobaric tags for relative and absolute quantitation
LM	Light microscopy
ME	Methyl eugenol
Ncc69	Sodium chloride cotransporter 69
Nano-LC-MS/MS	Nanoscale liquid chromatography coupled to tandem mass spectrometry
OBPs	Odorant-binding proteins
ODE	Odorant-degrading enzymes
ODPs	Odorant-degrading proteins
ORs	Odorant receptors
Orco	Odorant receptor co-receptor
OSNs	Olfactory sensory neurons
PBS	Phosphate buffered saline
Ped	Pedicel
Pka-R2	cAMP-dependent, regulatory subunit type 2
PKC	Protein kinase C
POBPA	Putative odorant-binding protein
Pp2A-29B	Phosphatase 2A at 29B
qRT-PCR	Quantitative real-time PCR
Rtn	Reticulon-like protein
RpS	Ubiquitin-40S ribosomal protein
Sc	Scape
SCX	Strong cation exchange
SDS	Sodium dodecyl sulfate
SDS-PAGE	Sodium dodecyl sulfate polyacrylamide gel electrophoresis
SEM	Scanning electron microscopy
SNMPs	Sensory neuron membrane proteins
SPoCk	Secretory Pathway Calcium atpase
(SPS)-MS	Synchronous precursor selection
TMT	Tandem mass tags
